# Correction: Kohsaka et al. Risk–Benefit Balance of Renin–Angiotensin–Aldosterone Inhibitor Cessation in Heart Failure Patients with Hyperkalemia. *J. Clin. Med.* 2022, *11*, 5828

**DOI:** 10.3390/jcm12072578

**Published:** 2023-03-29

**Authors:** Shun Kohsaka, Suguru Okami, Naru Morita, Toshitaka Yajima

**Affiliations:** 1 Department of Cardiology, Keio University School of Medicine, Tokyo 160-8582, Japan; 2 Cardiovascular, Renal, and Metabolism, Medical Affairs, AstraZeneca K.K., Osaka 530-0011, Japan

## Error in Figure

In the original publication [1], there was a mistake in Figure 3 as published. Some prescription numbers and percentages in ACEi, ARB and MRA were wrong. The corrected Figure 3 appears below. The authors state that the scientific conclusions are unaffected. This correction was approved by the Academic Editor. The original publication has also been updated.

## Figures and Tables

**Figure 3:**
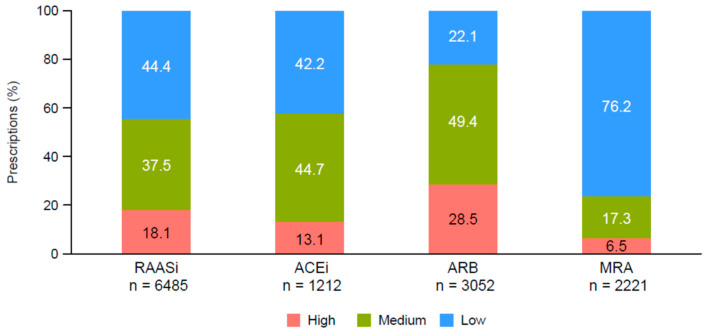
RAASi treatment according to dosage category (high, medium, or low) at the index date. Percentages were calculated using the total number of prescriptions. Abbreviations: ACEi, angiotensin-converting enzyme inhibitor; ARB, angiotensin receptor blocker; MRA, mineralocorticoid receptor antagonist; RAASi, renin–angiotensin–aldosterone system inhibitor.
