# Supplementary material for: Risk–Benefit Balance of Renin–Angiotensin–Aldosterone Inhibitor Cessation in Heart Failure Patients with Hyperkalemia
Source: J Clin Med. 2022 Sep 30;11(19):5828. doi: 10.3390/jcm11195828 (PMC9572691; doi:10.3390/jcm11195828)

Supplemental Material

Risk-benefit balance in cessation of renin–angiotensin–aldosterone inhibitors for heart failure patients presenting with hyperkalemia: A Japanese nationwide database analysis

Shun Kohsaka, MD[1], Suguru Okami, PhD[2], Naru Morita, MD, PhD[2], Toshitaka Yajima, MD, PhD[2]

[1]Department of Cardiology, Keio University School of Medicine, Tokyo, Japan

[2]Cardiovascular, Renal, and Metabolism, Medical Affairs, AstraZeneca K.K., Osaka, Japan

Corresponding author

Toshitaka Yajima

E-mail: Toshitaka.Yajima@astrazeneca.com

Table of contents

	Page
**Table S1.** Definitions of high-risk subgroups	2
**Table S2.** List of comorbidities	3
**Table S3.** Dosage categories for renin–angiotensin–aldosterone system inhibitors	4
**Table S4.** Definitions of clinical outcomes	5
**Table S5.** Propensity score-matched covariates	6
**Table S6.** Proportion of patients with RAASi dose reduction within 1 year of index date	7
**Figure S1.** RAASi and MRA cessation rates within 12 months of the index date<br>(A) All combinations of RAASi therapies. (B) All combinations including MRAs.	8

Table S1. Definitions of high-risk subgroups

Subgroup	Definition
Chronic kidney disease	Defined as either a diagnosis of chronic nephritic syndrome (ICD-10 code: N03), glomerular disease (N05-N08), chronic kidney disease/chronic renal failure (N18-N19), diabetic nephropathy (E102, E112, E122, E132, E142), or hypertension with renal failure (I120, I13), or the presence of an average eGFR of <60 mL·min^{-1}·1.73 m^{-2}<br>CKD stage 1: eGFR ≥90 mL·min^{-1}·1.73 m^{-2}, stage 2: eGFR 60-89 mL·min^{-1}·1.73 m^{-2}, stage 3a: eGFR 45-59 mL·min^{-1}·1.73 m^{-2}, stage 3b: eGFR 30-44 mL·min^{-1}·1.73 m^{-2}, stage 4: eGFR 15-29 mL·min^{-1}·1.73 m^{-2}, and stage 5: eGFR <15 mL·min^{-1}·1.73 m^{-2}
Diabetes mellitus	Defined as a diagnosis of DM (E10-E14)
Heart failure	Defined as a diagnosis of heart failure (I50, I110)
Hypertension	Defined as a diagnosis of hypertension (I10-I15)

Abbreviations: CKD, chronic kidney disease; DM, diabetes mellitus; eGFR, estimated glomerular filtration rate; ICD-10, International Classification of Diseases 10th revision

Table S2. List of comorbidities

Condition	ICD-10 code
Myocardial infarction	I21; I22; I23; I24
Peripheral vascular disease	I70; I71; I72; I73; I74; I77
Cerebrovascular disease	I60-I69; G45
Chronic pulmonary disease	J40-J47; J60-J67; J684; J701; J703; J841; J920; J961; J982; J983
Moderate to severe liver disease	B150; B160; B162; B190; K704; K72; K766; I85
Atrial fibrillation or atrial flutter	I48
Valvular heart disease	I00-I02; I05-I09; I34; I35; I36; I37; Q20-Q25
Alcoholism-related or other substance-abuse related disorders	T36-T65; F10-F19; G312; G612; G721; I426; K292; K860; K70; R780; T51; Z714; Z721
Acute kidney injury	N17
Sepsis	A021, A207, A227, A241, A267, A282, A327, A394, A400-A403, A409-A415, A418-A419, A427, A548, B007, B349, B377, D71, I301, I330, J020, J209, J950, L029, L080, M8699, O080, O753, O85, O883
Gastrointestinal bleeding	K250, K252, K254, K256, K260, K262, K264, K266, K284, K290, K571, K573
Gastrointestinal perforation	K251, K252, K255, K256, K261, K265, K266, K285, K570, K572
Peripheral oedema	R600

Abbreviation: ICD-10, International Classification of Diseases 10th revision

Table S3. Dosage categories for renin–angiotensin–aldosterone system inhibitors

RAASi therapy	Target maximum dose, mg/day	High-dose, mg/day	Medium-dose (70%), mg/day	Low-dose (30%), mg/day
**Angiotensin-converting enzyme inhibitors**				
Captopril	150	150	105	45
Enalapril	10	10	7	3
Lisinopril	20 (HF=10)	10	7	3
Trandolapril	2	2	1.4	0.6
Alacepril	100	100	70	30
Benazepril	10	10	7	3
Cilazapril	2	2	1.4	0.6
Derapril	120	120	84	36
Imidapril	10	10	7	3
Perindopril	8	8	5.6	2.4
Quinapril	20	20	14	6
Temocapril	4	4	2.8	1.2
**Angiotensin II receptor blockers**				0
Candesartan	12 (HF=8)	8	5.6	2.4
Losartan	100	100	70	30
Valsartan	160	160	112	48
Azilsartan	40	40	28	12
Irbesartan	200	200	140	60
Olmesartan	40	40	28	12
Telmisartan	80	80	56	24
**Mineralocorticoid receptor antagonists**				
Eplerenone	100 (HF=50)	50	35	15
Spironolactone	100	100	70	30

Abbreviations: HF, heart failure; RAASi, renin–angiotensin–aldosterone system inhibitor

Table S4. Definitions of clinical outcomes

Outcome	Definition
In-hospital death	Based on death information in the hospital discharge summary
Hospitalization for MI, arrhythmia, or cardiac arrest	Hospitalizations with ICD-10 codes of I21, I22, I23, I44, I45, I46, I47, I48, or I49
Hospitalization due to HF	ICD-10 code I50 or I110 as the main reason for hospitalization
Introduction of renal replacement therapy	Presence of a national receipt code for dialysis or renal transplant

Abbreviations: HF, heart failure; ICD-10, International Classification of Diseases 10th revision

Table S5. Propensity score-matched covariates

**Covariates**
Age, sex, index year
Length of follow-up
Non RAASi_yes
Loop or Thiazide _yes
Drugs for dyslipidemia_yes
Drugs for DM_yes
CKD stage
HTN_yes
Class of antihypertensive drugs
DM_yes, HbA1c
Inotropes use_YES
MRA use_YES
CCI score
Other comorbidities of interest
Had a hospitalization with ≥3 days LOS before the index date

Abbreviations: CCI, Charlson Comorbidity Index; CKD, chronic kidney disease; DM, diabetes mellitus; HbA1c, haemoglobin A1c; HTN, hypertension; LOS, length of stay; MRA, mineralocorticoid receptor antagonist; RAASi, renin–angiotensin–aldosterone system inhibitor

Table S6. Proportion of patients with RAASi dose reduction within 1 year of index date

	Patients with prescription at index date	Number of patients with dose reduction			
		Overall	High → Low	Medium → Low	High → Medium
**Any RAASi (ACEi, ARB or MRA)**					
n	5059	427	33	264	130
% of patients with prescription at index date	100	8.4	0.7	5.2	2.6
**ACEi**					
n	1211	72	5	52	15
% of patients with prescription at index date	100	5.9	0.4	4.3	1.2
**ARB**					
n	3037	221	19	106	96
% of patients with prescription at index date	100	7.3	0.6	3.5	3.2
**ACEi and/or ARB**					
n	4111	290	24	157	109
% of patients with prescription at index date	100	7.1	0.6	3.8	2.7
**MRA**					
n	2220	144	9	113	22
% of patients with prescription at index date	100	6.5	0.4	5.1	1.0

Abbreviations: ACEi, angiotensin-converting enzyme inhibitor; ARB, angiotensin receptor blocker; MRA, mineralocorticoid receptor antagonist; RAASi, renin–angiotensin–aldosterone system inhibitor

Figure S1. RAASi and MRA cessation rates within 12 months of the index date

(A) All combinations of RAASi therapies. (B) All combinations including MRAs.

Abbreviations: ACEi, angiotensin-converting enzyme inhibitor; ARB, angiotensin receptor blocker; MRA, mineralocorticoid receptor antagonist; RAASi, renin–angiotensin–aldosterone system inhibitor

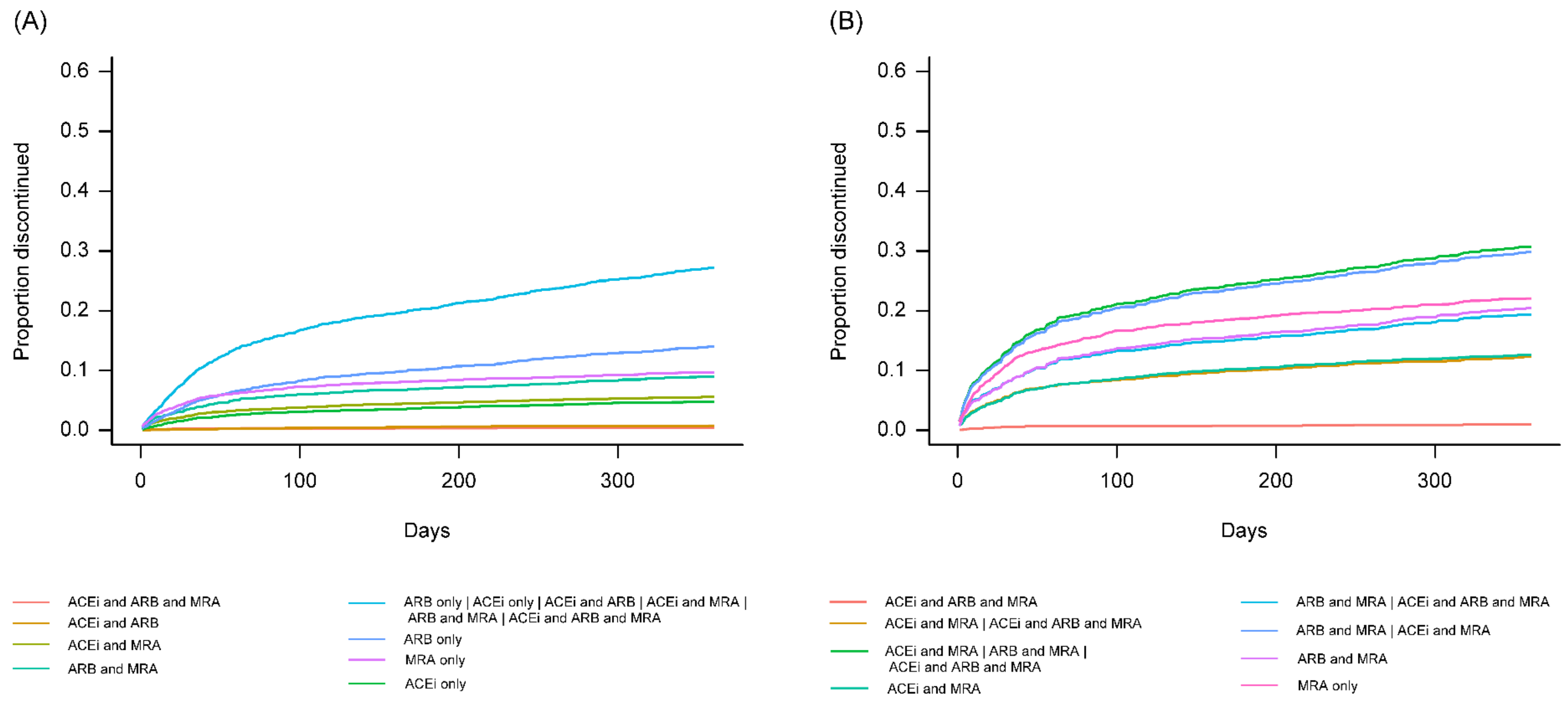

Supplement: Supplementary file 1 [file jcm-11-05828-s001.zip › jcm-1918447-supplementary.pdf]
